# MAO-B Elevation in Mouse Brain Astrocytes Results in Parkinson's Pathology

**DOI:** 10.1371/journal.pone.0001616

**Published:** 2008-02-20

**Authors:** Jyothi K. Mallajosyula, Deepinder Kaur, Shankar J. Chinta, Subramanian Rajagopalan, Anand Rane, David G. Nicholls, Donato A. Di Monte, Heather Macarthur, Julie K. Andersen

**Affiliations:** 1 Buck Institute for Age Research, Novato, California, United States of America; 2 Basic Science Research, Parkinson's Institute, Sunnyvale, California, United States of America; 3 Department of Pharmacological and Physiological Science, Saint Louis University School of Medicine, St. Louis, Missouri, United States of America; Mental Health Research Institute of Victoria, Australia

## Abstract

Age-related increases in monoamine oxidase B (MAO-B) may contribute to neurodegeneration associated with Parkinson's disease (PD). The MAO-B inhibitor deprenyl, a long-standing antiparkinsonian therapy, is currently used clinically in concert with the dopamine precursor L-DOPA. Clinical studies suggesting that deprenyl treatment alone is not protective against PD associated mortality were targeted to symptomatic patients. However, dopamine loss is at least 60% by the time PD is symptomatically detectable, therefore lack of effect of MAO-B inhibition in these patients does not negate a role for MAO-B in pre-symptomatic dopaminergic loss. In order to directly evaluate the role of age-related elevations in astroglial MAO-B in the early initiation or progression of PD, we created genetically engineered transgenic mice in which MAO-B levels could be specifically induced within astroglia in adult animals. Elevated astrocytic MAO-B mimicking age related increase resulted in specific, selective and progressive loss of dopaminergic neurons in the substantia nigra (SN), the same subset of neurons primarily impacted in the human condition. This was accompanied by other PD-related alterations including selective decreases in mitochondrial complex I activity and increased mitochondrial oxidative stress. Along with a global astrogliosis, we observed local microglial activation within the SN. These pathologies correlated with decreased locomotor activity. Importantly, these events occurred even in the absence of the PD-inducing neurotoxin MPTP. Our data demonstrates that elevation of murine astrocytic MAO-B by itself can induce several phenotypes of PD, signifying that MAO-B could be directly involved in multiple aspects of disease neuropathology. Mechanistically this may involve increases in membrane permeant H_2_O_2_ which can oxidize dopamine within dopaminergic neurons to dopaminochrome which, via interaction with mitochondrial complex I, can result in increased mitochondrial superoxide. Our inducible astrocytic MAO-B transgenic provides a novel model for exploring pathways involved in initiation and progression of several key features associated with PD pathology and for therapeutic drug testing.

## Introduction

Monoamine oxidase B (MAO-B) is found in the brain primarily in non-neuronal cells such as astrocytes and radial glia [1]–[3]. Its levels are known to increase with age and in association with neurodegenerative disease in both humans and mice [4]–[7]. Substrate oxidation by the enzyme is accompanied stoichiometrically by the reduction of oxygen to H_2_O_2_ [8], [9]. It has been postulated that age-related increases in MAO-B activity may contribute to cellular degeneration in the brain due to corresponding increases in the production of this reactive oxygen species (ROS)[10]. Although MAO-B is expressed primarily in astrocytes and not directly within dopaminergic cells, H_2_O_2_ has a high membrane permeability and therefore it can induce toxic effects not only within the cell of origin, but also in neighboring cells [11]. The area of the brain preferentially impacted in Parkinson's disease (PD), the substantia nigra (SN), contains high levels of MAO-B positive astrocytes which are themselves somewhat protected against the effects of H_2_O_2_ due to the fact that they contain high levels of both GSH and glutathione peroxidase which act in concert to detoxify H_2_O_2_ within cells [11]–[15]. Neurons, which contain significantly lower levels of these protective components, are particularly vulnerable to this mild oxidizing agent [16]–[18]. This suggests that H_2_O_2_ produced within astrocytes by MAO-B may be either broken down to H_2_O within these cells or may diffuse to vulnerable nearby cells such as dopaminergic neurons [19]. MAO-B activity levels have been found to be doubled in the SN in Parkinson's disease, and to correlate with the percentage of dopaminergic SN cell loss [20]. This suggests that increases in MAO-B levels could play a role in subsequent dopaminergic cell death.

MAO-B-catalyzed ROS production has been suggested to contribute to an age-related increase in mitochondrial damage particularly in the SN [21]. Previous *in vitro* studies from our laboratory demonstrated that increases in H_2_O_2_ generation as a consequence of inducible increases in MAO-B levels results in selective inhibition of mitochondrial complex I activity which impacted on mitochondrial function within cultured dopaminergic cells [22]. Selective reductions in complex I activity are associated with PD and selective inhibition of complex I via systemic administration of either rotenone or 1-methyl-4-phenyl-1,2,3,6-tetrahydropyridine (MPTP) results in patterns of morphological damage in rodents similar to that observed in the human Parkinsonian midbrain[23], [24]. MAO-B is responsible for conversion of MPTP to MPP^+^ within astrocytes from where MPP^+^ can diffuse extracellularly. From there, it can be selectively transported into dopaminergic neurons via the dopamine transporter and elicit inhibitory effects on mitochondrial complex I activity [25].

There are numerous reports demonstrating the neuroprotective effects of the MAO-B inhibitors deprenyl or selegiline in Parkinsonian animal models although in many cases neuroprotection has been attributed to either antioxidant or antiapoptotic properties of the parent compounds or their metabolites [26]–[28]. Several multi-center studies including DATATOP, addressing the efficacy of selegiline administration in Parkinson's disease (PD) patients have concluded that selegiline both delayed and reduced the requirement of L-DOPA supplementation in early stages of symptomatic PD [29]–[31] but had no effect on the mortality rate [32]. Although deprenyl was found to be ineffective in slowing or halting the ultimate mortality in clinical PD it was given to patients only after symptoms were present which is known to coincide with the presence of an already extensive (∼60%) midbrain dopaminergic cell loss [33]. These studies suggest that inhibition of MAO-B activity at this advanced stage in the disease is ineffective but this does not necessarily prove that MAO-B elevation is not a factor in initial neuropathology associated with the disorder and MAO-B inhibitors if given pre-symptomatically might not be protective. In order to test the hypothesis that elevations in MAO-B can directly contribute to pathologies observed in PD and to better understand the possible mechanisms underlying its effects on these parameters, we created genetically engineered mouse lines in which MAO-B levels can be inducibly increased specifically within astrocytes in adult animals. This allowed us to bypass any impact of its increased expression during the developmental time period. We report here that astrocytic elevation of MAO-B in adult mice recapitulates many of the pathological hallmarks associated with human PD and that these effects can be prevented by treatment with the selective MAO-B inhibitor deprenyl or the antioxidant EUK189.

## Results

### Creation of transgenic mouse lines in which astrocytic MAO-B activity can be inducibly increased in adult animals

**Figure 1:**
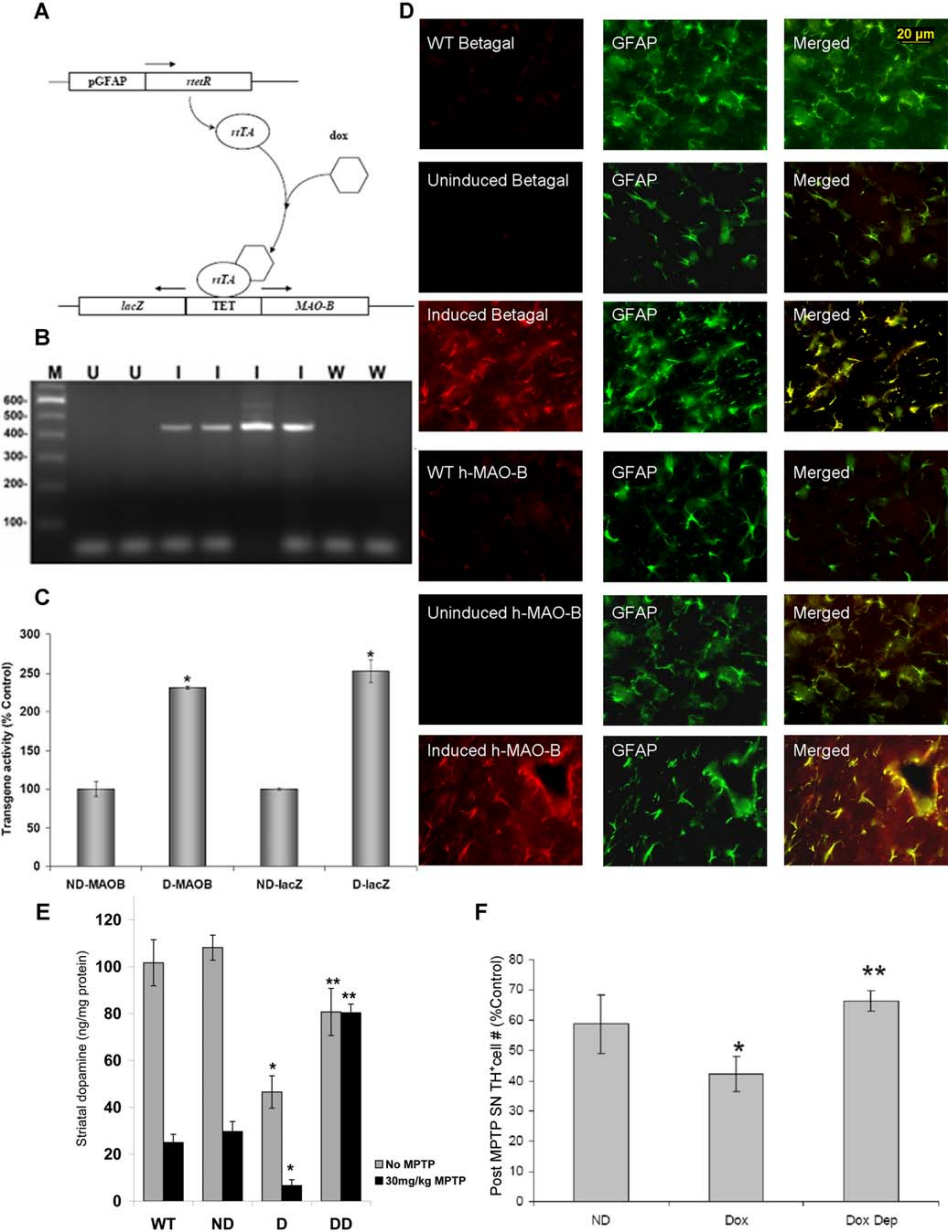
Characterization of transgenic mice that inducibly express human MAO-B specifically within astrocytes. A. Schematic of transgenic constructs utilized to create transgenic mice with inducible elevation of human MAO-B (h-MAO-B) expression specifically within astrocytes via the bacterial tetracycline regulatory system. A bacterial reverse tetracycline response gene (*rtetR)* was expressed constitutively via a mouse glial fibrillary acidic protein promoter (pGFAP). The resulting gene product, rtTA, a tet-responsive transactivator protein, is activated only in the presence of doxycycline (dox) and is able to bind to a tetracycline operator (TET) sequence in the promoter of a second transgene, resulting in induction of expression of both h-*MAO-B* and bacterial beta-galactosidase (lac Z) from the bidirectional promoter. B. Human MAO-B cDNA transgene is expressed in the brains of transgenic mice following dox induction. Inducible transgene mRNA expression (a 448 bp amplicon) was visualized by RT-PCR from RNA isolated from whole brains of uninduced (U), dox-induced (I) transgenic mice and wild type littermates (W). C. Enzymatic activities of h-MAO-B and lacZ transgenes in whole brain lysates from uninduced (ND) and dox-induced (D) transgenic animals. n = 3 animals per condition run in triplicate; * p<0.01. Data is presented as percent ND control. ND lacZ activity was calculated to be 11.9±0.54 nmoles ONPG converted/mg protein/hour and wild type C57BL6 lacZ activity (not shown) to be 11.3±0.13 nmoles ONPG converted/mg protein/hour respectively. Brain MAO-B activity in tissue isolated from the uninduced transgenic (ND) was calculated to be 14.6±0.9 nmoles β-PEA converted/mg protein/hour and in tissues from wild type C57BL6 mice (not shown) to be 14.2±0.23 nmoles β-PEA converted/mg protein/hour. D. Dox-inducible expression of human MAO-B (h-MAO-B) and lacZ is localized within GFAP^+^ astrocytes. GFAP immunostaining is shown in green, h-MAO-B is red, and merged in yellow in uninduced (No DOX) and dox-induced (DOX) animals. Scale bar applies to all images. E. Loss in striatal dopamine (ST DA) content is exacerbated in dox-induced transgenics following a single MPTP injection of 30 mg/kg body weight. Striatal dopamine was estimated from wild type (W), uninduced transgenics (ND), induced transgenics (D), and dox-induced transgenics co-treated with deprenyl (Ddep). Grey bars without MPTP, black bars after a single injection of 30 mg/kg MPTP. Data is expressed as ng dopamine/mg striatal protein. * p<0.01 D vs. ND, ** = p<0.01 Ddep vs. ND. F. Loss in dopaminergic tyrosine-hydroxylase-positive (TH^+^) neurons in the substantia nigra (SN) after a single injection of MPTP (30 mg/kg) is exacerbated in dox-induced mice. Uninduced transgenic mice (ND), induced transgenics (Dox), and induced transgenics co-treated with 10mg/kg deprenyl (Doxdep). Data is presented as percent of non-MPTP treated ND controls where SN TH^+^ cell numbers are estimated at 13,318±475 TH^+ ^neurons in untreated ND mice (WT values, not shown, were 13,699±325). n = 3 animals per condition run in triplicate; * p<0.05.

Double transgenic mouse lines were generated in our laboratory that constitutively express a reverse tetracycline responsive transactivator (rtTa) protein specifically within astrocytes via a glial fibrillary acidic protein promoter (pGFAP). Upon addition of the tetracycline derivative doxycycline (dox), rtTa binds to a tetracycline responsive bidirectional promoter (TET) and induces simultaneous expression of both wildtype human MAO-B cDNA (GenBank accession number NM 000898) and the marker protein bacterial beta-galactosidase (lacZ) (Figure 1A). Expression of the two co-transgenes was induced in our studies via inclusion of dox in the normal mouse chow, and visualized by RT-PCR (Figure 1B). Whole brain extracts from induced animals with the highest levels of expression exhibited an approximate 2.5-fold increase in MAO-B and lacZ activities versus uninduced controls (Figure 1C); increased MAO-B activity was completely inhibited by deprenyl treatment (data not shown). This high-expressing line was used for all subsequent studies. Immunohistochemistry using antibodies specific to MAO-B demonstrated that the human MAO-B was exclusively expressed within (GFAP) astroglia throughout the brain and only following dox treatment (Figure 1D).

### Elevation in astrocytic MAO-B results in increased conversion of MPTP to MPP^+^ and significant increases in toxin-induced dopaminergic SN cell loss

To validate that our transgenic model expresses functionally active MAO-B, we assessed its vulnerability to acute systemic MPTP administration. Since conversion of MPTP to MPP^+^ is catalyzed by MAO-B, we predicted that elevation in functional astrocytic MAO-B should result in increased striatal MPP^+^ levels and exacerbation of selective MPTP-induced dopaminergic SN neurodegeneration. Induced and uninduced transgenic mice and wildtype C57Bl6 littermates were treated with 30 mg/kg MPTP and striatal MPP^+^ levels measured via HPLC (Table 1). At 90 minutes post MPTP injection, striatal MPP^+^ levels were 1.5-1.8-fold that observed in either uninduced transgenic or wildtype controls. This increase in striatal MPP^+^ was accompanied by a significant decrease in striatal dopamine levels and dopaminergic SN cell numbers in induced transgenics compared to controls seven days following MPTP treatment (Figures 1E & 1F). MAO-B elevation alone was found to result in ∼60% reduction in striatal dopamine levels (Figure 1E) which was exacerbated further by MPTP. Elevations in astrocytic MAO-B in our transgenics resulted in exacerbation of MPTP-induced neurotoxicity suggesting that astrocytic MAO-B increases had the expected functional effect.

**Table 1:** Striatal MPP^+^ levels.

MPP^+^ levels	
	MPP^+ ^(ng/mg protein)
Dox	182±3.4
No dox	118±2.3
WT	100±3.5

MPP^+^ levels were measured in striatal tissues collected from wildtype (WT), uninduced (No dox) and dox-induced (Dox) transgenic mice 90 min post-MPTP injection (30 mg/kg) via HPLC with electrochemical detection. MPP^+^ was extracted from tissues homogenized in the presence of 0.4N perchloric acid and measured against a known standard. Values are presented as ng MPP^+^/mg protein , n = 3 per treatment condition.

**Figure 2:**
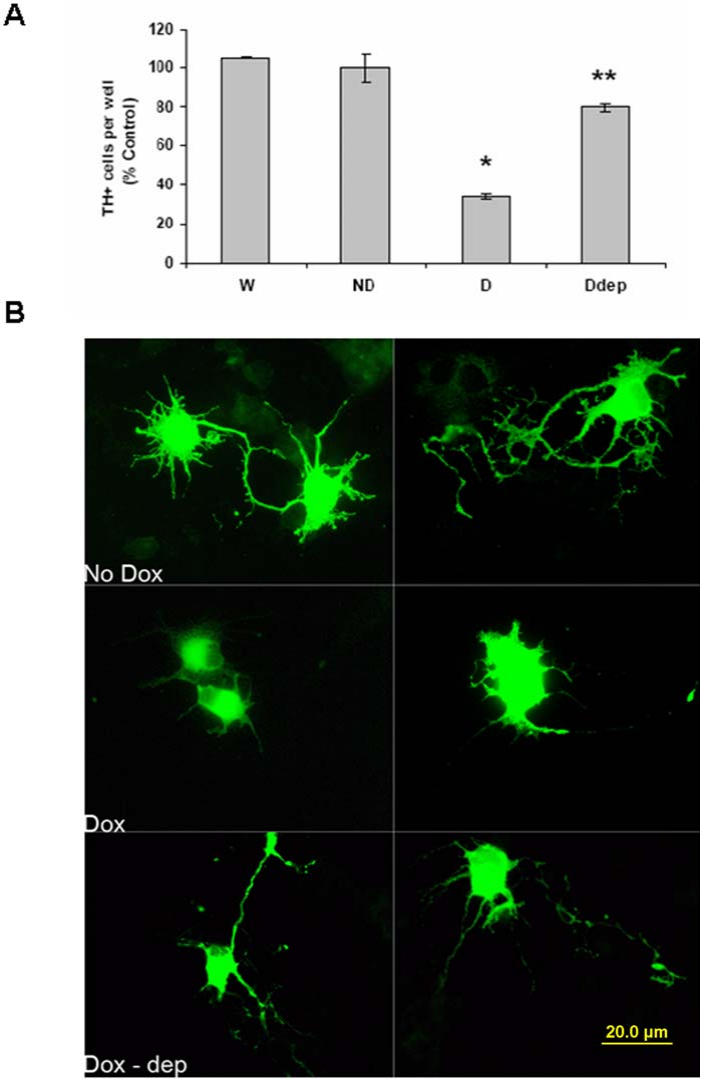
Inducible elevation of astrocytic MAO-B levels in mesencephalic cultures isolated from transgenic mice results in TH^+^ cell loss which is prevented by co-treatment with deprenyl. A. TH^+^ cell counts in uninduced mesencephalic cultures isolated from either wildtype littermates (WT), uninduced transgenics (ND) or transgenic cultures induced with 40 µg/ml doxycycline for 24 hrs in the absence (D) or presence of 10^-6^M deprenyl (Ddep). Counts are expressed as % WT values which were estimated to be 300±15 TH^+^ neurons. Cell counts were performed in a minimum of three different wells and 4 separate fields in each well. * = p<0.01 compared to ND; **p>0.01 compared to Dox. B. Representative micrographs of the TH^+^ cells obtained by TH^+^ immunofluorescence in uninduced (No Dox), dox-induced (Dox) and dox-induced mesencephalic cultures treated with deprenyl (Doxdep). Note the truncated processes and shrunken dopaminergic cell morphology in the induced cultures versus uninduced and deprenyl-treated induced cultures.

Astrocytic MAOB elevation in mesencephalic cultures isolated from inducible MAO-B lines was found to result in dopaminergic neurodegeneration. Mesencephalic cultures were prepared from day-14 embryos from transgenic mice and wildtype littermates. Mixed neuronal/glial mesencephalic cultures were grown on coated plates for 3 days and transgenic MAO-B activity induced in a portion of the transgenic cultures by addition of 40 µg/ml dox. Dox was added to wildtype cultures as a negative control. Twelve hours following dox addition, immunocytochemistry performed on dox-induced transgenic cultures revealed a significant loss in the number of tyrosine hydroxylase positive (TH^+^) cells compared to controls even in the absence of toxin addition that was attenuated by deprenyl co-treatment (Figure 2A). To verify that loss of TH immunostaining represents actual preferential loss of dopaminergic cells, we counted the number of cells within the cultures which take up the fluorescent dopamine analogue ASP^+^ (4-(4-(dimethylamino)styryl)-N-methylpyridinium iodide, Molecular Probes) as a measure of cells with functional dopamine transporter activity. We found a similar percentage loss of ASP^+^ cells (∼80%, 44±22 versus 244±68 uninduced per field). This demonstrates that the loss in TH^+^ cell numbers is not simply a consequence of selective loss of this particular marker. In addition to increased dopaminergic cell loss, the remaining dopaminergic neurons were observed to undergo significant loss of their neurite processes which became stunted and shrunken in size. Deprenyl co-treatment attenuated not only the extensive dopaminergic cell loss observed following induction of MAO-B within cultured astrocytes but also the observed morphological dopaminergic neurite alterations (Figure 2B).

### Elevations of astrocytic MAO-B *in vivo* results in selective dopaminergic SN cell loss

**Figure 3:**
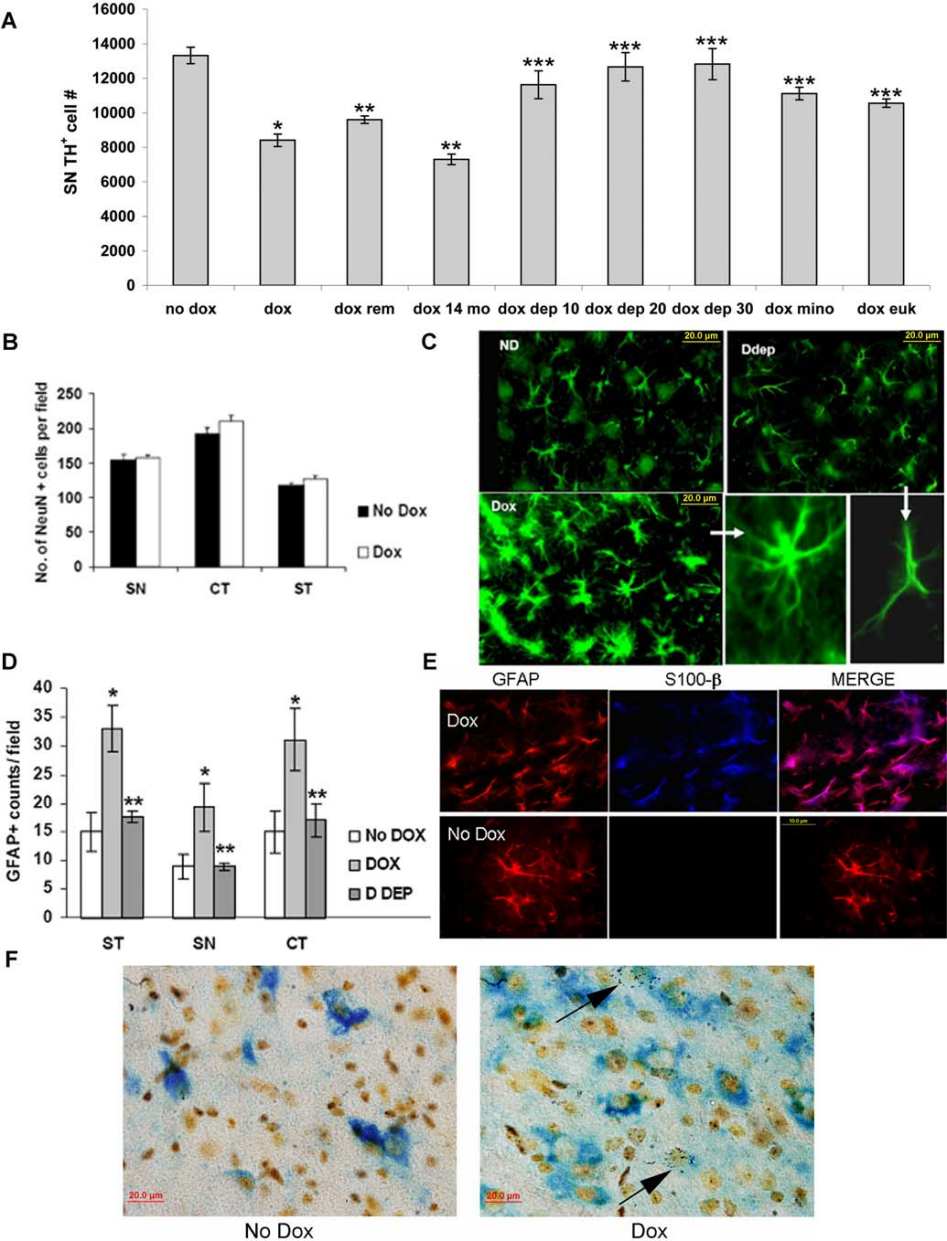
Inducible elevation in astrocytic MAO-B results in selective, age-related SN dopaminergic neurodegeneration and global astrocyte activation even in the absence of neurotoxin. A. TH^+^ SN cell counts from uninduced transgenics (no dox) versus induced transgenics at 3-4 months (dox) , following dox removal (dox rem) at 14 months of age (dox 14 mo), and induced co-treated with deprenyl at 10-30 mg/kg (dox dep 10-30), the anti-inflammatory agent minocycline at 10 mg/kg (dox mino) or the anti-oxidant Euk189 at 10 mg/kg in young animals. Data is expressed as total number of SN TH^+^ cells numbers per animal; *p<0.01 compared to ND, ** p< 0.05 compared to Dox, and *** p< 0.05 compared to dox. B. Neuronal (NeuN^+^) counts from uninduced (No Dox, filled bars) versus dox-induced transgenics (Dox, open bars) performed in equivalent nigral (SN), cortical (CT), and striatal (ST) tissue sections. Values represent average cell numbers per field in three separate fields per section, three sections per animal. N = 5 animals per group. C. Representative micrographs demonstrating cortical astrocytic activation in dox-induced (Dox) versus uninduced (ND) animals and dox-induced animals co-treated with deprenyl (Ddep). Astrocytes from induced transgenics displayed enhanced GFAP staining and increased branching indicative of astrocyte activation (higher magnification in lower middle panel); deprenyl treatment resulted in reduced branching similar to that observed in uninduced controls (higher magnification in lower right-hand panel). D. Astrocytic (GFAP^+^) counts from uninduced (No DOX, open bars) versus dox-induced transgenics (DOX, light grey bars) and mice dox-induced in the presence of deprenyl co-treatment (D DEP, dark grey bars) performed in equivalent striatal (ST), nigral (SN), and cortical (CT) tissue sections. Values represent average cell numbers per field at 40×magnification in three separate fields per section, three sections per animal. N = 5 animals per group; *p>0.05, ** p<0.1. E. Representative micrograph demonstrating increased expression of the astrocytic activation marker S-100β (blue) in GFAP^+^ cells in the transgenic cortex following dox induction. GFAP-labeled astrocytes (red) in induced animals display increased S-100β staining (pink) versus uninduced. F. Neurodegeneration of TH^+^ cells as assessed by silver staining. SN sections from uninduced (No Dox) and induced (Dox) animals were processed for TH^+^ immunoreactivity visualized by alkaline peroxidase (blue) and further processed for silver staining. A black punctate staining was observed in and around dying TH^+^ neurons (arrows) in Dox but not No Dox fields.

To assess the impact of astrocytic MAO-B elevation on its own *in vivo* on the nigrostriatal system, stereological dopaminergic cell counts were performed on dox-fed transgenic mice versus controls. A two week induction period in 2–3 month old transgenics resulted in an approximate 40% loss in dopaminergic SN cell numbers compared to either untreated transgenics or wildtype littermates; this loss was prevented by deprenyl co-treatment (Figure 3A). Moreover, removal of dox from the animal feed after the initial two weeks for three weeks did not result in a reversal of TH^+ ^counts to normal levels. Astrocytic MAO-B elevation in older (14 mo.) transgenics resulted in an even more pronounced dopaminergic SN cell loss (∼50%) than that observed in the younger animals. In contrast, total neuronal numbers in the SN as assessed by NeuN staining did not differ between dox-treated transgenics versus untreated controls nor were neuronal cell numbers in the striatum or cortex impacted by astrocytic MAO-B elevation (Figures 3B), even though it occurred throughout the brain and resulted in a general and widespread astrogliosis as evidenced by GFAP immunocytochemistry in these same brain regions (Figures 3C, D). GABAergic SN cell counts also revealed no difference in dox-induced versus non-induced animals further suggesting that the observed dopaminergic cell loss in this brain region was selective (data not shown). GFAP immunostaining revealed profuse branching as normally observed following astroglial activation and increased astroglial brain numbers following dox treatment which were inhibited by deprenyl. Levels of S100-β, a marker for activated astrocytes, were also increased in transgenic astroglia following dox induction (Figure 3E).

We performed silver staining to verify that selective loss of TH^+^ immunostained cells in the SN following dox induction coincided with increased neurodegeneration in this brain region (Figure 3F). This data along with the dox removal results suggests that the decrease in TH^+^ SN cells is not merely due to a reversible selective loss of the TH^+^ marker but constitutes an irreversible dopaminergic SN neurodegeneration.

### Astrocytic MAO-B elevations result in increased mitochondrial oxidative stress in dopaminergic SN neurons

**Figure 4:**
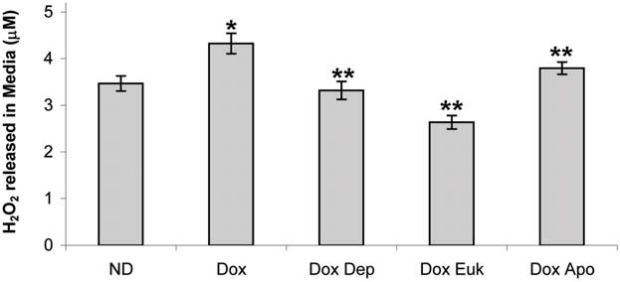
Elevation in astrocytic MAO-B results in increased extracellular hydrogen peroxide (H_2_O_2_). Extracellular H_2_O_2 _concentrations were measured in media isolated from uninduced (ND) versus dox-induced (Dox) mesencephalic cultures in the absence and presence of 10 µM deprenyl (Dox Dep), 30 µM EUK189 (Dox Euk) or 500 nM apocynin (Dox Apo) using the Amplex Red assay. H_2_O_2_ concentrations were quantified against a standard H_2_O_2_ curve. Measurements were made on three separate media aliquots harvested prior to immunostaining of cultures; *p<0.01 compared to ND, ** p<0.05 compared to ND.

Based on our data, global increases in astrocytic MAO-B appeared to result in a preferential neurodegeneration of dopaminergic neurons suggesting that dopamine itself may be involved in the neurodegenerative process. Recent work in isolated brain mitochondria [34] demonstrated that dopamine oxidized to dopaminochrome (DACHR) can remove electrons from the auto-oxidizable site in mitochondrial complex I forming dopaminochrome radical. Dopaminochrome radical can in turn react with O_2_ present in the mitochondria converting it to O_2_^−^ in concert with re-reduction of the dopaminergic radical, setting up a redox cycling event. This would be manifest as an increase in mitochondrial O_2_^−^ levels and ROS-induced complex I inhibition within the dopaminergic neurons which could result in their selective demise. We hypothesized that membrane-permeant H_2_O_2_ produced by increased MAO-B activity within astrocytes could interact with intercellular dopamine and produce selective degenerative effects on dopaminergic neurons via a similar mechanism. To first test for the ability of astrocytes with elevated MAO-B activity to produce increased levels of extracellular ROS, we measured H_2_O_2_ generated in the culture medium in mesencephalic cultures isolated from our inducible MAO-B transgenics following dox induction. H_2_O_2_ levels released into the media in dox-induced cultures were significantly higher than those produced from untreated cultures (Figure 4). Co-treatment with deprenyl prevented the increase in extracellular H_2_O_2_ as did treatment with the antioxidant superoxide dismutase-catalase mimetic compound EUK-189. Co-treatment with apocynin, an NADPH oxidase inhibitor, resulted in a partial reduction in the H_2_O_2 _levels indicating that microglial activation contributes to the ROS increase although it is not clear whether this is directly due to astroglial activation or a secondary effect of dopaminergic cell death in the cultures. Iba1^+^ cells (a 17 kDa calcium binding protein expressed in microglia) were found to constitute 3% of the total cell population and their numbers were increased by ∼17% following dox induction (data not shown).

**Figure 5:**
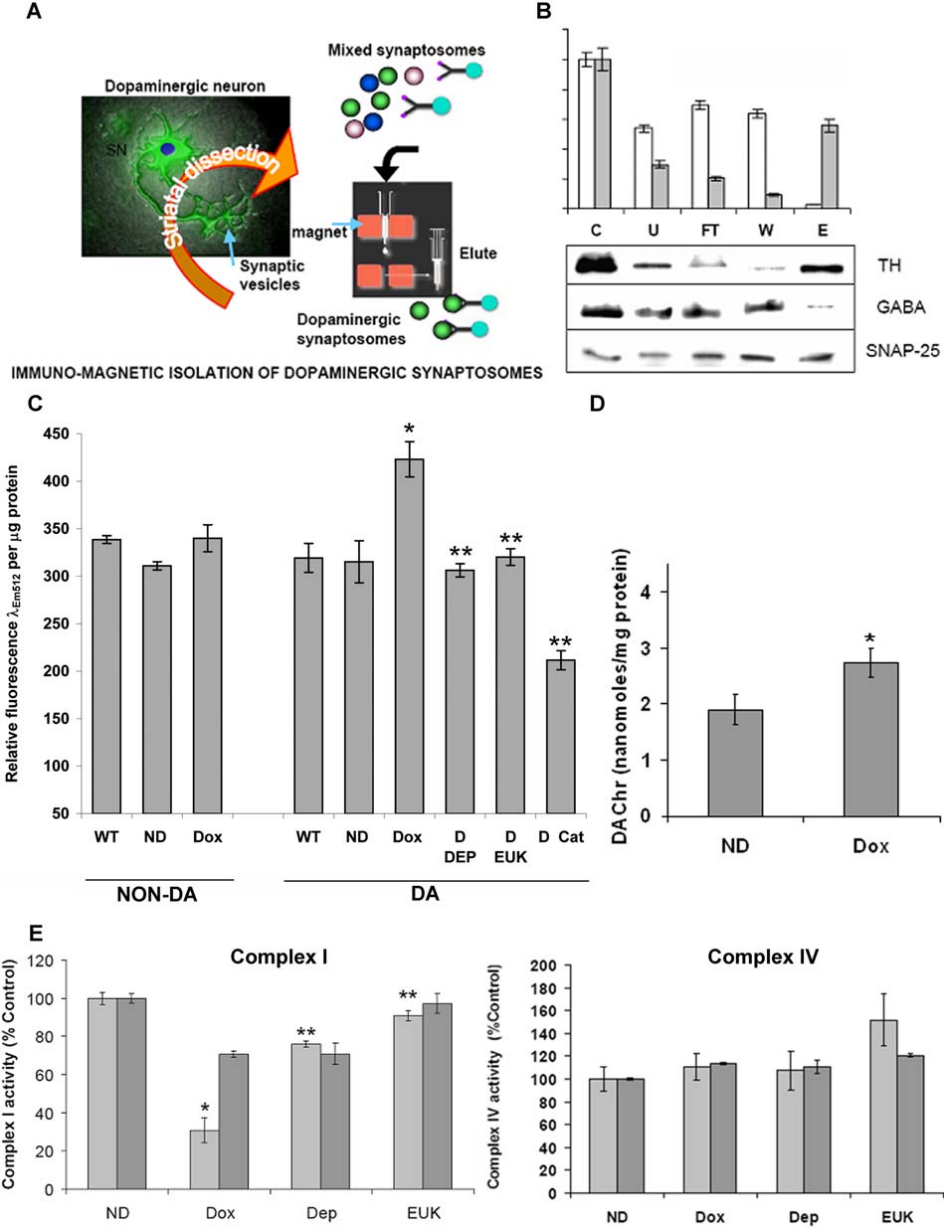
Effects of elevations of astrocytic MAO-B on H_2_O_2_ and dopaminochrome (DAChr) levels, and mitochondrial complex I versus IV activities in isolated ST dopaminergic versus non-dopaminergic synaptosomes. A. Schematic of novel immunomagnetic technique utilized to for isolate ST dopaminergic versus non-dopaminergic synaptosomes. Synaptosomes were prepared from freshly dissected striata and the dopaminergic population rapidly isolated via immunoprecipitation with anti-DAT antibody following by separation by a magnetic particle-conjugated secondary antibody running through a strong magnetic column. Elution of the DA synaptosomes was obtained via removal of the magnetic field. Flow-through contains striatal non-dopaminergic synaptosomes used as negative controls in subsequent biochemical experiments. B. Densitometric quantitation of western blot analyses of fractions from immunomagnetic isolation of ST synaptosomes probed with TH, GABA or SNAP-25 antibodies. Levels of Dopaminergic TH (grey bars) versus Non-dopaminergic GABA (white bars) synaptosomes were assessed using SNAP-25, a general synaptic protein, as a loading control. Control (C), unbound fraction (U), flow-through (FT), wash (W) and eluate (E). Representative blots are shown in the lower panel. C. Elevation in astrocytic MAO-B results in increased H_2_O_2_ within dopaminergic ST synaptosomes. H_2_O_2_ levels were estimated 3 hrs following tail vein injection of DCFDA into either wildtype littermates (WT), uninduced (ND) or dox-induced (Dox) transgenics, or induced transgenic animals co-treated with either deprenyl (D DEP), EUK189 (D EUK) or 1 mg/ml catalase (Dox Cat). DCF fluorescence was examined at an excitation wavelength of 488 nm and emission at 512 nm. Data is reported normalized to microgram synaptosomal protein. N = 3; *p<0.005 versus ND, ** = p<0.01 versus Dox. D. Elevation in astrocytic MAO-B results in increased ST DAChr levels. DAChr levels were estimated in ST samples from uninduced (ND) versus dox-induced (Dox) transgenic mice via HPLC. Values are presented as picomoles DAChr/mg protein.* p<0.02, n = 3. E. Elevation in astrocytic MAO-B results in decreased complex I but not complex IV activity in ST dopaminergic synaptosomes. Complex I and complex IV activities were measured in isolated ST dopaminergic versus non-dopaminergic synaptosomes from uninduced (ND), dox-induced (Dox) and induced transgenic animals co-treated with either deprenyl (Dep) or EUK-189 (EUK). N = 3–5 animals per group and a total of 3 separate experiments, values are expressed as mean±SD; * p<0.01 compared to ND, ** p>0.05 compared to ND. CI activity was calculated to be 4.0 versus 3.3 µM NADH/min/mg protein in ND striatal dopaminergic and non-dopaminergic synaptosomes, respectively. CIV activity was calculated to be 139 versus 109 µM ferrocytochrome c/minute/mg protein in ND striatal dopaminergic and non-dopaminergic synaptosomes, respectively. Activities of WT DA striatal synaptosomes was calculated to be 4.4 µM NADH/min/mg protein (CI) and 130 µM ferrocytochrome c/minute/mg protein (CIV); dox feeding of the WT animals did not alter the activities. Note: addition of doxycycline has been reported to interfere with mitochondrial protein translation including CI thereby affecting enzyme activity [62], however in addition to lack of effect on CI activity in WT animals +/− dox, we also find no change in CI subunit protein levels in WT mice as a consequence of dox treatment as assessed via immunoprecipitation/gel electrophoretic analyses (data not shown).

Dopaminergic neurons constitute only a minor portion (∼ 5%) of cells making up the SN (unpublished observations). In contrast to the SN, the striatum (ST) is larger, easier to dissect out, and at least 10–15% of the striatal nerve terminals originate from SN dopaminergic neurons [35]–[37]. In order to achieve meaningful data as to the impact of astrocytic MAO-B elevation on H_2_O_2_ levels and mitochondrial function selectively within dopaminergic nigrostriatal neurons, we chose to enrich for striatal dopaminergic nerve terminal (synaptosomal) populations using antibody against DAT via a modified immunomagnetic bead approach (Fig. 5A,B). Synaptosomes in the flow-through fractions were non-dopaminergic (GABA^+^) and therefore used as negative controls in our subsequent biochemical analyses.

H_2_O_2_ levels were measured in isolated ST dopaminergic synaptosomes following injection of DCF into the tail vein of dox-induced versus uninduced and wildtype littermate controls as modified from Andrews et al. [38]. H_2_O_2_ levels was found to be elevated in isolated ST dopaminergic synaptosomes following increased astrocytic MAO-B elevation; this was found to be preventable by co-treatment of animals with either deprenyl or EUK-189 (Fig. 5C). Catalase treatment of the dopaminergic synaptosomes from the induced animals reduced the fluorescence to below uninduced levels indicating that the DCF fluorescence is due to H_2_O_2_. In conjunction with elevations in H_2_O_2_ within nerve terminals originating from dopaminergic SN neurons, we found an elevation in striatal DACHR. DACHR is a stable derivative of dopamine quinone, the major species produced by dopamine oxidation [39] (Figure 5D). Along with increases in DACHR levels, we found a preferential reduction in rotenone-inhibitable complex I (CI) activity in the ST dopaminergic versus non-dopaminergic synaptosomes (Figure 5E). In contrast, no change in complex IV activity was observed in either population following dox-induction (Figure 5E). Loss of CI activity was attenuated in dopaminergic synaptosomes isolated from dox-induced animals that had been co-treated with deprenyl or EUK-189.

**Figure 6:**
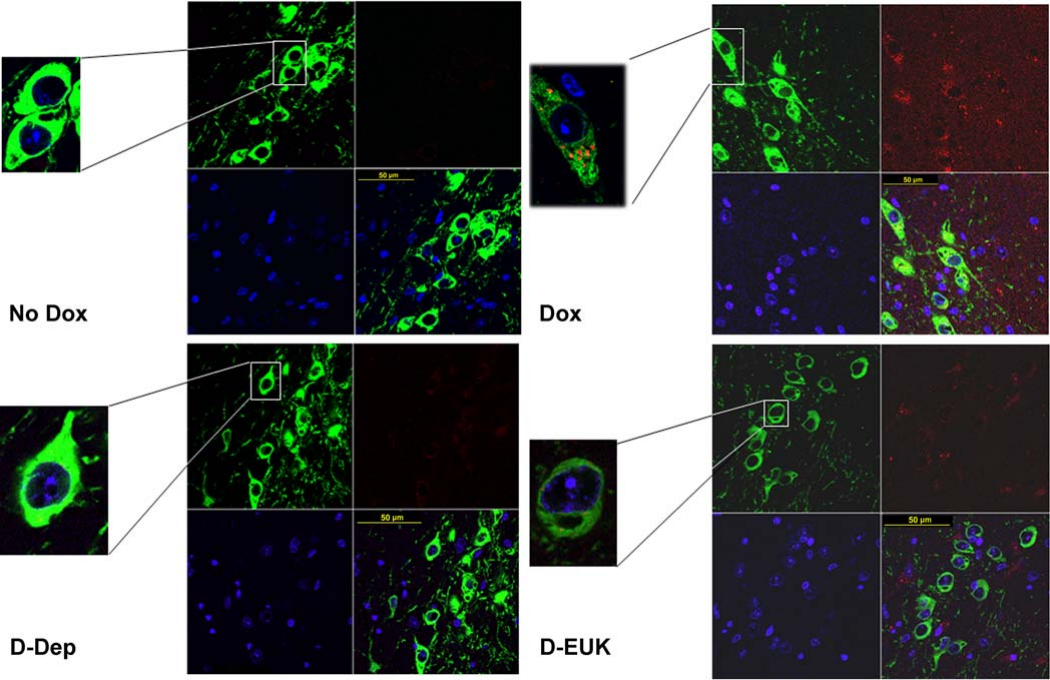
Elevation in astrocytic MAO-B results in increased mitochondrial superoxide levels within dopaminergic SN mitochondria. Representative confocal micrographs illustrating increased merged (yellow) mitosox red fluorescence (red) in the SN and striatum of dopaminergic neurons following tail vein injection, fixation and TH immunochemistry (green) in uninduced (No Dox) versus induced transgenic mice (Dox) which is prevented in the presence of co-deprenyl (DEP) or EUK-189 (EUK) treatment. Nuclei are visualized via DAPI staining (blue).

To assess whether increased dopaminergic H_2_O_2 _and DACHR levels following astrocytic MAO-B elevation in vivo resulted in corresponding increases in mitochondrial O_2_^− ^levels within dopaminergic cells as previously demonstrated in vitro by Zoccarato and colleagues [34], animals were administered the superoxide indicator mitosox red via tail vein injection[38]. Dox-induced transgenics displayed elevated presence of mitosox red staining within mitochondria in both TH^+^ fibers in the striatum and in TH^+ ^neurons within the SN versus uninduced controls (Figure 6, Table 2). Increases in mitosox red fluorescence within the dopaminergic SN neurons were prevented by co-treatment with either deprenyl or EUK189 (Figure 6, Table 2). Significantly, dopaminergic SN cell loss was found to be attenuated in the presence of co-treatment with not only deprenyl, but also EUK189 suggesting that oxidative stress is an important component of dopaminergic demise (Figure 3A).

**Table 2:** Superoxide in the SN dopaminergic neurons.

	Mean pixel intensity	Total pixel fluorescence
	per cell	per cell
ND	10.8±2.0	101,983±17,686
D	24.2±3.6	223,526±28,225
DD	10.2±2.1	91,937±19,107
DEUK	15.4±4.0	126,757±23,798

Confocal quantitation of relative mitosox-red fluorescence intensity in SN DA neurons in untreated (ND) or dox-treated (D) transgenic mice in the absence or presence of deprenyl (DD) or EUK-189 (DEUK). Confocal images from immunofluorescent sections of brains were obtained following various treatment regimes and subjected to intensity measurements using the IMARIS software suite (Bitplane AG, Zurich, Switzerland) on selected cells from multiple sections from each treatment. Data is presented both as mean pixel intensity and total red fluorescent pixels (mitosox red) per cell.

### Increases in astrocytic MAO-B activity results in increased local microglial activation in the SN

**Figure 7:**
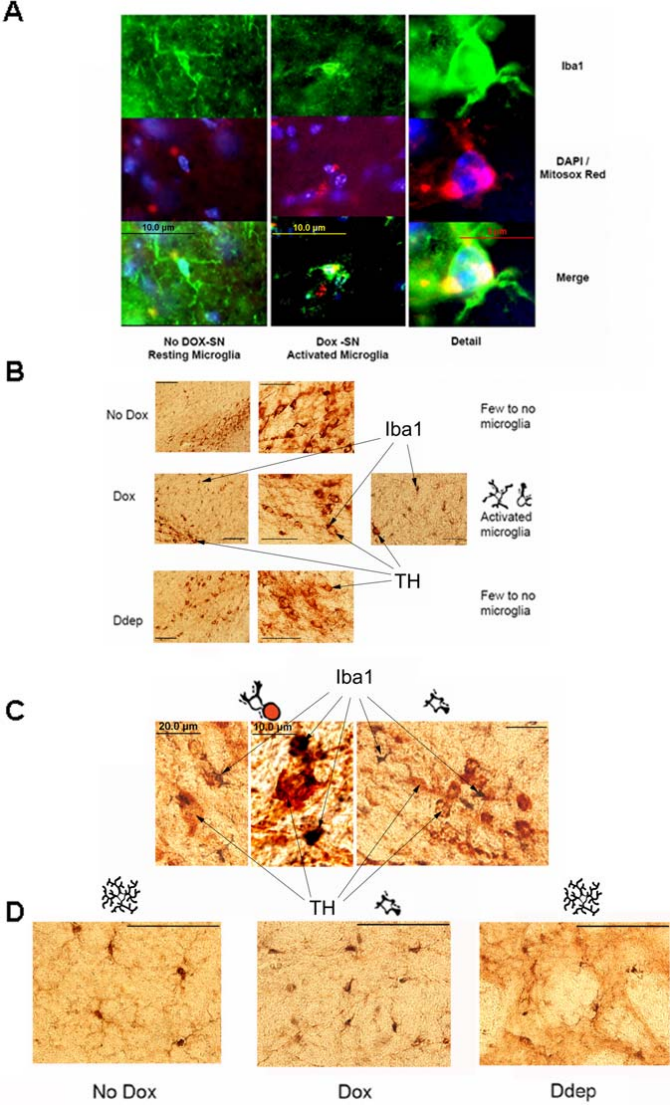
Global elevation in astrocytic MAO-B results in local SN microglial activation. A. Mitosox staining of local SN microglia following dox treatment reveals increased mitochondrial superoxide levels within these cells. Microglia were immunostained with Iba1 (green) antibody in sections of brain fixed 3 hrs following tail injection of mitosox red. Confocal detail shows mitochondrial mitosox red fluorescence within an activated microglial cell body following dox induction. B. Microglial activation occurs following dox induction within the SN. Microglial activation as assessed by Iba1 immunochemistry (black, DAB-nickel staining) in SN sections from uninduced (No Dox), induced (Dox), and dox-induced co-deprenyl-treated transgenics (Ddep). Dopaminergic (TH^+^) neurons are labeled in brown (DAB). Note that few activated microglia are visible in No Dox and Ddep treated mice versus in the Dox panel where several stages of microglial activation (resting, activated, withdrawing, and motile stages) can be visualized. Scale bar equals 50 µM unless indicated otherwise. C. Higher magnification of representative SN Iba1 immunochemistry demonstrating stages of microglial activation (activated, withdrawing, and motile stages are indicated by cartoons). Cortical microglia were found to be in a ramified or resting stage in all the conditions (supplementary figure S1). D. Representative Iba1 immunochemistry in ST sections demonstrating increased microglial activation following dox induction. Shown are ST sections from uninduced (No Dox) versus dox-induced (Dox) or dox-induced, co-deprenyl treated (Ddep) transgenics. Note the presence of numerous retracted processes in the microglia in the Dox panel versus presence of ramified microglia in the No Dox and Ddep panels.

Data from our mitosox red studies not only suggested that SN dopaminergic neurons display an increase in oxidative stress following astrocytic MAO-B elevation, but also in the activated local SN microglial cells (Fig. 7A). This is likely a secondary effect subsequent to dopaminergic SN neurodegeneration triggering local microgliosis. Degeneration of SN dopaminergic neurons in PD has been observed to be accompanied by local microglial activation [40]. It has also been noted post-mortem in both humans and primates exposed to MPTP [41], [42]. It is detected in the chronic mouse rotenone model prior to appearance of the dopaminergic lesion [43] and during selective SN dopaminergic neurodegeneration in the spontaneous weaver mouse mutant [44]. We performed immunochemistry to evaluate microglial activation in our induced MAO-B transgenics via Iba1 immunostaining [45]. Prior to activation, microglia normally exhibit a highly ramified morphology. In response to an activating signal, microglia begin to withdraw their ramified branches and to extend new protrusions. Next they begin migration through the tissue, engulfing cells [46]. Astrocytic MAO-B expression was found to lead to local microglial activation in both the SN and the striatum (Figure 7 B–D) but not the cortex (supplement Figure S1). Unactivated ramified microglia were found in the uninduced nigrostriata and cortex; activated microglia with reduced branching in locomotory stages were only present within the SN and striatum of induced MAOB transgenics. In some cases, the microglia were in a high motile state with minimal processes or were in close contact with TH^+^ neurons, possibly in the phagocytic stage (Figure 7C). Microglial activation was prevented by treatment with deprenyl (Figure 7B). Co-treatment of animals with an inhibitor of microglial activation, minocycline, prevented dopaminergic SN cell death suggesting that microglial activation plays a major role in dopaminergic neurodegeneration in this model (Figure 3A).

### Increases in astrocytic MAO-B activity results in decreased locomotor movement

**Figure 8:**
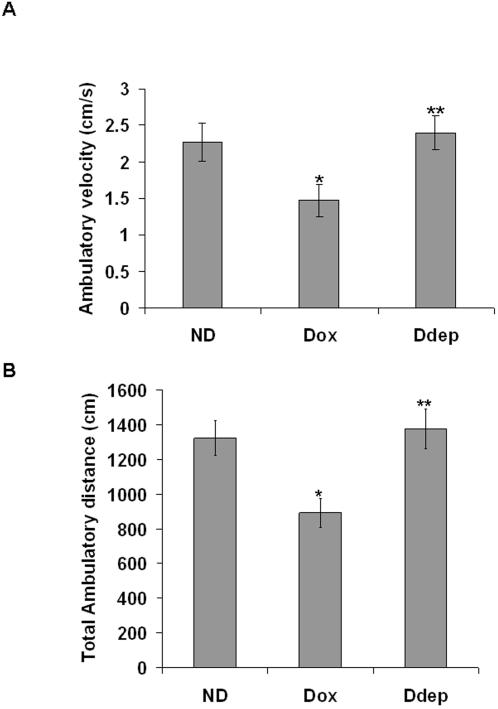
Elevation in astrocytic MAO-B results in loss of ambulatory movement in MAO-B transgenics. Total ambulatory velocity (A) and ambulatory distance (B) following two weeks of dox administration. Data is presented as average ambulatory velocity calculated over a 10 minute observation period and total ambulatory distance is for that same time interval. N = 5 mice per condition; experiments were run in triplicate. * p<0.01 compared to ND, ** p>0.05 compared to ND. Values are given for untreated (ND), dox-treated (Dox), and dox, deprenyl co-treated mice. Wildtype untreated littermates had an ambulatory velocity of 2.37±0.45 cm/s and a total ambulatory distance moved of 1082±101 cm.

Finally, astrocytic increases in MAO-B in our model were found to correlate with a significant inhibition of locomotor function. Open field analysis of dox-treated mice revealed a significant difference in locomotor behavior in induced versus uninduced transgenics (Figure 8A, B). After treatment with dox alone for two weeks, induced mice displayed a ∼32–35% decrease in locomotor activity in comparison to uninduced littermates. In contrast, deprenyl-treated induced mice were completely unaffected.

## Discussion

Increased brain MAO-B levels have been hypothesized to play a role in neuropathies associated with PD [47]–[51] however direct proof of a causative role has been thus far lacking. In this study, we demonstrate that elevations in astrocytic MAO-B levels results in a relatively selective loss of dopaminergic SN neurons and the severity of this loss appears to be age-dependent. It is not clear why there was not a more dramatic increase in SN DA cell loss with age in the MAO-B transgenics. Normally, cell loss coincides with an age-related increase in MAO-B enzyme activity. Our results suggest that perhaps the elevation of MAO-B to aging levels in the young animals was sufficient on its own to produce significant cell loss and that the additional stress of an aging brain contributed marginally (yet significantly) to this effect. Observed cell loss was accompanied by increased mitochondrial oxidative stress and selective decreases in mitochondrial complex I activity in these cells along with local microglial activation all of which we assume contribute to subsequent cell death. These pathological alterations were found to correlate with a significant decrease in locomotory behavior. Our data, taken in total, demonstrates that elevations in levels of astrocytic MAO-B activity results in several of key pathological hallmarks of Parkinson's disease.

**Figure 9:**
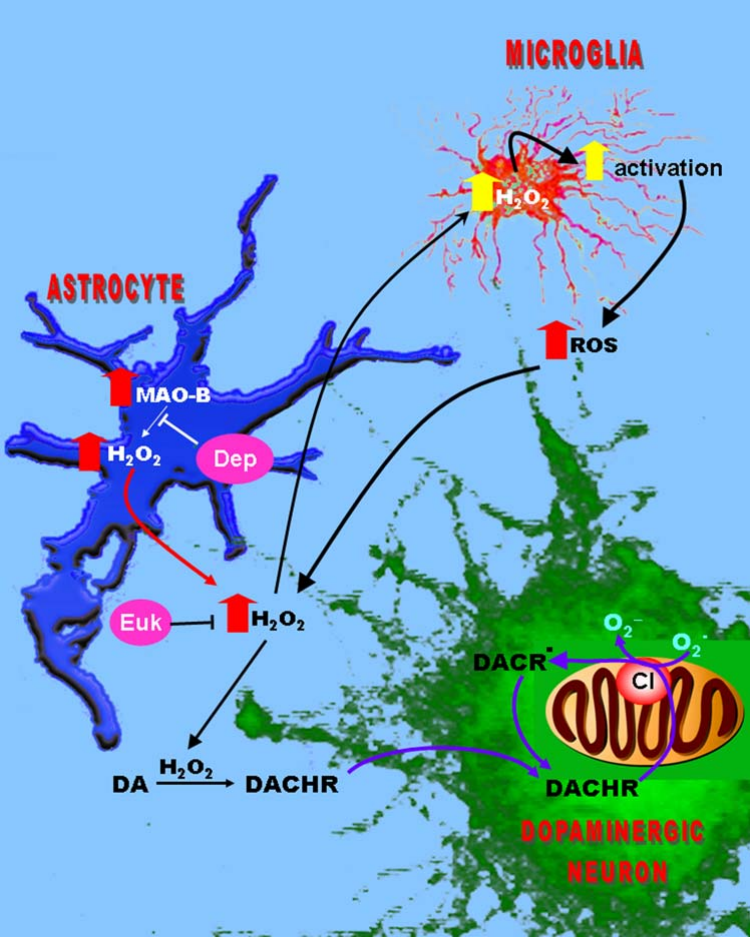
A schematic representation of the events occurring upon the elevation of glial MAO-B. Increased astroglial levels of MAO-B results in elevation of H_2_O_2_ levels; this can be prevented by deprenyl. Astroglia are themselves protected from H_2_O_2_ due to relatively high levels of glutathione-gluathione peroxidase versus neurons. H_2_O_2 _can diffuse out of astroglia and into neighboring cells including local dopaminergic neurons and microglia; this can be prevented by EUK. Dopamine can be oxidized by H_2_O_2_ through dopamine quinone formation to stable dopaminochrome (DACHR). DACHR can extract an electron from the auto-oxidizable site of mitochondrial complex I forming DACR radical and impacting selectively on complex I function. Due its high affinity for molecular oxygen, DACR radical can pass an electron on to this molecular species resulting in its re-reduction to DACHR and increased formation of mitochondrial superoxide radical. MAO-B generated H_2_O_2_ can also stimulate local microglia; microglial activation can be greatly secondarily enhanced within the SN due to dopaminergic cell demise which can also exacerbate ROS production and impact on local dopaminergic neurons.

ROS produced by MAO-B-expressing astrocytes can be released into the extracellular environment and, due to its high membrane permeability, diffuse into neighboring cells. Within dopaminergic neurons, it can oxidize dopamine to DACHR which can interact with electrons at the auto-oxidizable site of mitochondrial complex I at a higher affinity than oxygen itself producing DACR radical. Electrons can then be transferred from DACR radical to oxygen resulting in re-reduction of DACR to DACHR and production of superoxide as part of an ongoing redox cycling event. These events are shown schematically in Figure 9. We have demonstrated in this current study that increased astrocytic MAO-B results in elevations in extracellular ROS levels and increased intracellular H_2_O_2_ within the terminals of SN dopaminergic neurons accompanied by increased dopamine oxidation to DACHR, selective mitochondrial complex I inhibition and elevations in mitochondrial superoxide levels. These events are prevented by either co-treatment with the MAO-B inhibitor deprenyl or the antioxidant compound EUK189 which also prevented subsequent dopaminergic SN cell loss.

Another prominent feature noted in our model was local microglial activation within the nigrostriatum. Dramatic local proliferation of microglial cells has been reported in the post-mortem PD brain as well as in various animal models of the disease [40], [43], [44], [52]–[54].Local microglial activation was found to be associated in our model with degenerating TH^+^ cells in both the SN and the striatum. It may be a secondary reaction to initiation of dopaminergic neurodegeneration. Significantly, dopaminergic SN cell loss is substantially attenuated by co-treatment of animals with the microglial activation inhibitor minocycline suggesting that microglial activation plays a significant role in dopaminergic demise associated with this model.

Taking in total, our data demonstrates a linkage between several separate pathological events associated with Parkinson's disease that have not necessarily been considered to be mechanistically connected including mitochondrial dysfunction, and microglial activation. This suggests that MAO-B may be a common initiator for these events and provides a novel model for exploring the mechanisms by which these events can occur in the context of the human condition.

## Materials and Methods

### Generation and analysis of transgenics with inducible astrocyte-specific elevation of MAO-B levels

Human MAO-B cDNA was cloned into pBIG (Clontech) at the PstI and SalI sites, placing the gene under transcriptional control of the bidirectional Tet-inducible promoter. A second construct was prepared which contained the 3.7 kb HinDIII-BamHI fragment of the mouse GFAP promoter cloned in EcoRI blunt ended pTetOn vector, driving expression of the Tet-responsive reverse transactivator rtTA. Transgenic C57Bl6 mice inducibly expressing human MAO-B selectively within astrocytes were produced by co-injecting the two transgenes into the male pronuclei of a fertilized egg prior to implantation into a pseudopregnant female. Subsequent founder mice were crossed with wildtype C57Bl6 and resulting litters genotyped by PCR using the primers 5′ACTGAACCCAAAGGCACAC3′ and 5′AACATGACAATGAAGGAGCTAC3′ for MAO-B, and 5′GCCTTTACCCATTACC3′ and 5′CCCGCTTATTTTTAATGC3′ for rTta. Littermates positive for both transgenes were mated to achieve homogeneous lines. Astroglial-specific transgene expression was induced by feeding animals doxycycline at 3000 ppm provided in pre-mixed Purina chow (Research Diets) for a three week period. Assuming a 5 g diet per day for a 30 g mouse, the dosage achieved was calculated to be equivalent to 0.5 g/kg/day. Total RNA was isolated from cortical brain tissue of wildtype uninduced and uninduced transgenic mice using Trizol (Invitrogen).according to manufacturer's instructions. cDNA was prepared using Superscript III reverse transcriptase kit (Invitrogen) and oligo-(dT) primers from 2 µg of total RNA. PCR was carried out with the forward and reverse primers corresponding to human MAO-B cDNA as above using Taq polymerase (Eppendorf). A 448bp amplicon indicates the induction of the h-MAO-B transcript. Positive transgene induction was also confirmed based on MAO-B and betagalactosidase positive-immunostaining within astrocytes of dox-induced transgenics using antibodies specific to hMAO-B and betagalactosidase within GFAP^+^ cells. Betagalactosidase and MAO-B enzyme activities were also performed in whole brain homogenates from induced versus uninduced transgenic lines [2], [55]. The line showing the highest level of induction was used for all subsequent studies. Mice were housed according to standard animal care protocols, fed *ad libitum*, kept on a 12 hr light/dark cycle, and maintained in a pathogen-free environment in the Buck Institute Vivarium. Animals used for subsequent studies were either young adults (2–6 months of age) or older animals (14 months of age) fed dox on the regime described above. Co-treatment with other agents involved intraperitoneal administrations of 10–30 mg/kg/day of deprenyl, minocycline or EUK-189 for two weeks.

### MPP^+^ measurements, striatal dopamine content cell counts and neurodegeneration in inducible astrocytic MAO-B transgenics versus controls +/- MPTP

Striatal tissue was harvested from dox versus untreated animals 90 minutes following injection of 30 mg/kg MPTP intraperitoneally, immediately immersed in ice cold 0.1 M perchloric acid and sonicated. MPP^+^ was measured by HPLC as previously described [56]. Dopamine levels were analyzed in striata dissected two weeks of induction or 24 hrs following MPTP injection post induction by HPLC followed by a 464 pulsed electrochemical detection (Neurochemistry Core, Center for Molecular Neuroscience, Vanderbilt University). Stereological cell counts were performed on immunostained brain sections from brains harvested two weeks after induction using either antibody against tyrosine hydroxylase (TH, Chemicon, 1∶500) followed by biotin-labeled secondary antibody and development using DAB (Vector Laboratories). TH^+^ cells were counted stereologically throughout the SNpc [56]. Counts for dox removal were performed on brains dissected after removal of dox from the feed for a three week period. Sections were cut at a 40 µm thickness, and every 4th section was counted using a grid of 100×100 µm. Dissector size used was 35×35×12 µm. Neuronal numbers were assessed following NeuN^+ ^(Chemicon, 1∶100) or GABA^+^ (Chemicon, 1∶1000) immunostaining and astrocytes via GFAP^+^ (DAKO, 1∶500) and s100-β (DAKO, 1∶200) immunostaining. Cells were counted in the SN, striata, and cortex in three independent sections per condition and ten fields per section. Neurodegeneration in the TH^+^ cells was visualized by silver staining (FD Neurotechnologies, Ellicott City, MD) according to the manufacturer's instructions using proprietary compounds after immunostaining the sections with antibody against tyrosine hydroxylase (TH, Chemicon, 1∶500) visualized with Vector Blue alkaline phosphatase (Vector labs). Dopaminochrome was measured from striatal samples by HPLC as per Ochs et al [39].

### Extracellular H_2_O_2_ levels and dopaminergic cell survival in mesencephalic cultures

Primary mixed cultures were prepared from the midbrain of 14-day-old mice embryos from MAO-B transgenics and WT controls. Tissue was digested in Neurobasal medium containing 30 U/ml papain and 20 µg/ml DNase at 37°C for 30 min and mechanically triturated. Dissociated cells were centrifuged at 500 × g, resuspended in growth medium (Neurobasal medium supplemented with 10% FBS, 2 mM glutamate, B25 supplement without antioxidants, 50 U/ml penicillin, 50 U/ml streptomycin and 50 ng/ml GDNF [57]), and plated on poly-d-lysine-coated 8 well chamber slides (BD-Biocoat) at a density of 10^5^ cells per ml. Mixed cultures were grown at 37°C for 3–5 days before induction with 40 µg/ml doxycycline for 12 hours as per [22]. Cells were co-treated with 10 µM deprenyl or 500 nM apocynin. H_2_O_2_ in the medium was assayed by Amplex Red per the manufacturer's instructions. Dopaminergic and microglial cell counts were performed following fixation of the cultured cells for 15 min with 4% formaldehyde following by TH^+^ (Chemicon) or Iba1^+^(WAKO USA, Richmond, VA) immunostaining or via uptake of ASP^+^ (4-(4-(dimethylamino)styryl)-*N*- methylpyridinium iodide) (Invitrogen), a fluorescent analogue of dopamine. 10 µM ASP^+^ was loaded on to the uninduced or induced cultures in the presence of 20 µM of desipramine to inhibit the norepinephrine transporter which would also take up the dye. ASP^+^ fluorescence (red) was visualized in the TH^+^ cells after immunostaining with anti-TH antibody and Alexa fluor 488 conjugated secondary antibody. Averages of manual double blind TH^+ ^or fluorescent counts of three wells of culture with the same treatment were collected; counts were performed in triplicate.

### Immuno-magnetic isolation of dopaminergic synaptosomes

Dopaminergic and non-dopaminergic striatal synaptosomes were isolated using a modified immuno-magnetic protocol [58]. Briefly, synaptosomes were prepared from dissected striatal tissue [59]. Dopaminergic synaptosomes were isolated using magnetic beads conjugated to anti-rabbit IgG (Miltenyi Biotech) after capturing them using a rabbit anti-DAT antibody (Chemicon). Under a magnetic field, bead-bound synaptosomes were passed through a column and washed; flow-through in the presence of the magnetic field yielded a fraction enriched in non-dopaminergic striatal synaptosomes. Elution performed in the absence of the magnetic field yielded fractions enriched in dopaminergic striatal synaptosomes. Equal quantities of various fractions of the synaptosomes isolated using DAT antibody including the starting material or control, the unbound fraction, the flow-through, the wash and the eluate were subjected to western analysis to assess relative dopaminergic versus non-dopaminergic synaptosomal purification. Western blots were performed using antibodies against TH (Chemicon 1∶500), GABA (Chemicon, 1∶1000) or SNAP-25 as a synaptic protein normalization control (Chemicon, 1∶1000). Band densities were quantified using a Chemimager (Alpha Innotech, San Leandro, CA). Approximately 94% synaptosomes in the eluate were dopaminergic according to densitometric evaluation of the western blot.

### H_2_O_2_ estimation in synaptosomes

Hydrogen peroxide levels were measured in the striatal dopaminergic and non-dopaminergic synaptosomes prepared as above from various groups of mice. Mice were injected in the tail vein with ∼200 µl of DCFDA (Calbiochem) which was freshly diluted 100 x with PBS from a stock of 100 mg/ml in DMSO as modified from Andrews et. al. [38]. The animals were sacrificed 3 hours later and synaptosomes were prepared as above. Hydrogen peroxide in the synaptosomes with or without 1mg/ml catalase for 30 min after preparation was visualized as DCF fluorescence at 488 nm excitation and 512 nm emission in a Spectramax Gemini fluorescence plate reader. The relative fluorescence was normalized to synaptosomal protein which was quantified using the Bradford reagent, Bio-Rad.

### Mitochondrial complex I and IV activities

Isolated synaptosomes were found to be physiologically viable for up to 3 hours. Complex I activities were assayed in isolated dopaminergic and non-dopaminergic synaptosomal fractions from dox-induced animals as rotenone-sensitive NADH dehydrogenase activity by measuring DCPIP (2,6-dichlorophenolindophenol) reduction in synaptosomal extracts following addition of 200 µM NADH, 200 µM decylubiquinone, 2 mM KCN, and 0.002% DCPIP in the presence and absence of 2 µM rotenone [60]. Complex IV activity was assayed as cytochrome c oxidase activity by observing the rapid (1–2s) oxidation of freshly reduced 40 µM ferrocytochrome c in a 10 mM K-PO_4_ buffer pH 7.2 containing 100 mM KCl, 0.025% maltoside at 30°C, at 550 nm, averaged over two concentrations per sample. Values for all assays were normalized/protein using BioRad reagent.

### Detection of mitochondrial superoxide *in vivo*

Mitosox Red (Molecular Probes) was used as an indicator to assess presence of superoxide within cellular mitochondria. 10 µg of Mitosox in 200 µl phosphate-buffered saline was injected into the tail vein of dox-induced transgenics versus controls. Animals were sacrificed 90 minutes later, brains rapidly dissected out and post-fixed overnight in 4% paraformaldehyde. Brains were sectioned at 30 µm using a cryostat. Cellular localization and site of Mitosox Red accumulation in the nigrostriatum was identified by immunofluorescence using anti-TH (Chemicon, 1∶500), anti-IBA1 (Wako, 1∶500) or anti-GFAP (DAKO, 1∶500) antibodies visualized with Fluorescein Avidin D. Mitochondrial localization was confirmed by immunoanalysis using an anti-ATP synthase-specific antibody. Fluorescent imaging was done using a Zeiss LSM510 NLO confocal microscope and images procured using LSM software. Quantification of colocalization and fluorescent intensity was determined using the IMARIS software suite (Bitplane AG, Zurich, Switzerland) on selected cells from multiple sections.

### Morphological analyses of astrocyte and microglial activation

Immunochemistry was performed on cryosections derived from the SN, striata, and CTX of fixed perfused brains from dox-induced transgenics versus controls. Astrocytic activation was assessed using primary antibodies against GFAP and s100-β. Microglial activation was detected using primary antibodies against Iba1. Horse radish peroxidase or avidin-conjugated secondary antibodies were used to detect primary antibodies following either DAB or Fluorescein/Texas Red staining.

### Locomotor behavioral assessment

Locomotor function was assessed using an automated Truscan photobeam apparatus (Colbourne Instruments, Allentown, PA) under illumination as previously described [61]. Animals were habituated to the apparatus for 10 min then data collected over a 10-min period followed by analysis using Truscan 99 software. Spontaneous horizontal movement was assessed in both control and dox treated versus untreated transgenic mice via both ambulatory velocity and distance in a 10-min period.

## Supporting Information

Figure S1 Microglial activation is largely absent in the cortex. Absence of detectable microglial activation in the cortical sections from untreated, dox-treated deprenyl co-treated animals. Iba1 immunocytochemistry reveals that cortical microglia are in a resting ramified stage in all the three types of treatments conditions in contrast to what occurs in the striatum and the substantia nigra (Figure 6). This suggests that activation in the nigrostriatum may largely be due to secondary effects as a consequence of SN dopaminergic demise. Two representative micrographs are provided from each treatment.
(10.35 MB TIF)
