# Correction: MAO-B Elevation in Mouse Brain Astrocytes Results in Parkinson's Pathology

**DOI:** 10.1371/annotation/3c37bef4-bb5e-4f1e-8551-01afc006df90

**Published:** 2012-08-06

**Authors:** Jyothi K. Mallajosyula, Deepinder Kaur, Shankar J. Chinta, Subramanian Rajagopalan, Anand Rane, David G. Nicholls, Donato A. Di Monte, Heather Macarthur, Julie K. Andersen

Figure 4A, published in December 2007 in Chinta et al., J. Neurosci. 27: 13997, was inadvertently reproduced as Figure 5A in this article. The authors apologize for this duplication. These figures concern a novel technique developed in our laboratory to isolate striatal dopaminergic synaptosomal populations from mouse brain and subsequently independently used as part of separate transgenic mouse studies described in these two publications. Permission has been granted by Journal of Neuroscience for the publication of this figure in the PLoS ONE article under a Creative Commons Attribution license.

